# 

**Published:** 1898-08-06

**Authors:** 


					THE H0^> pita L. "The standard of highest purity."?Lancet, August 6tit, 1898.
CADBURY'S barfk HUM Is** CADBURY'S
cocoa mm. jm cocoa
absolutely pure. no or alkalies used.
No. 619. Vol. XXIV. Satubday,
August 6th, 1898.
[-Suhsraription Temi,] pRICE TWOPENCE.

				

